# Revisiting Antiarrhythmic Drug Therapy for Atrial Fibrillation: Reviewing Lessons Learned and Redefining Therapeutic Paradigms

**DOI:** 10.3389/fphar.2020.581837

**Published:** 2020-11-09

**Authors:** Meng Geng, Andrew Lin, Thao P. Nguyen

**Affiliations:** Division of Cardiology, Department of Medicine, The Cardiovascular Research Laboratory, David Geffen School of Medicine at UCLA, Los Angeles, CA, United States

**Keywords:** atrial fibrillation, a-fib, afib, antiarrhythmic, antiarrhythmic drug therapy, paroxysmal AF, persistent AF, permanent AF

## Abstract

Since the clinical use of digitalis as the first pharmacological therapy for atrial fibrillation (AF) 235 years ago in 1785, antiarrhythmic drug therapy has advanced considerably and become a cornerstone of AF clinical management. Yet, a preventive or curative panacea for sustained AF does not exist despite the rise of AF global prevalence to epidemiological proportions. While multiple elevated risk factors for AF have been established, the natural history and etiology of AF remain incompletely understood. In the present article, the first section selectively highlights some disappointing shortcomings and current efforts in antiarrhythmic drug therapy to uncover reasons why AF is such a clinical challenge. The second section discusses some modern takes on the natural history of AF as a relentless, progressive fibro-inflammatory “atriomyopathy.” The final section emphasizes the need to redefine therapeutic strategies on par with new insights of AF pathophysiology.

## Introduction

Atrial fibrillation (AF) is the most common sustained arrhythmia with rising global prevalence and a 37% lifetime risk. In AF, the chaotic rapid atrial excitation renders atrial contractions ineffective and ventricular contractions irregularly irregular. The resulting hemodynamic disturbances in AF lead to deleterious clinical consequences. Blood stagnation and flow turbulence in the fibrillatory atrium increase the risk of stroke by five-fold (Wolf et al., 1991), heart failure by three-fold (Stewart et al., 2002), dementia by two-fold (Ott et al., 1997), and death by two-fold (Stewart et al., 2002). The rapid ventricular response in AF often causes symptoms and if left untreated, prolonged tachycardia can induce cardiomyopathy over time (Nerheim et al., 2004). Even when the ventricular response is normal or slow, AF can still cause symptoms that limit exercise tolerance and reduce quality of life. While there was just under half a million of AF-related hospitalizations in the United States in 2010 (Patel et al., 2014), this number has been increasing with each subsequent year (Nisar et al., 2016). AF treatment costs $26 billion annually in the United States alone (Kim et al., 2011; Rozen et al., 2018) and this financial burden will continue to rise as AF soars to epidemic proportions.

Since the first recognition of AF in animals 392 years ago by William Harvey in 1,628 (McMichael, 1982) and since the first ECG depiction of AF in humans 113 years ago by Willem Einthoven in 1906 (Einthoven, 1906; Silverman, 1994), several pathogenic mechanisms have been proposed (Burstein and Nattel, 2008; Greiser et al., 2011). Elevated risk factors for AF were established (January et al., 2014), with advancing age as the most prominent of all (Benjamin et al., 1994; Schnabel et al., 2015). In the United States, extended longevity from medical advances has led to the projection of up to 15 million patients diagnosed with AF by 2050 (Miyasaka et al., 2006; Colilla et al., 2013). Modifiable risk factors for developing AF within 10 years (Schnabel et al., 2009) include hypertension, diabetes mellitus (Patel et al., 2014), coronary artery disease (Peigh et al., 2020), heart failure, obesity (Thacker et al., 2013), and obstructive sleep apnea (Youssef et al., 2018). Non-modifiable AF risk factors include valvular heart disease (Banerjee et al., 2019), hypertrophic cardiomyopathy (Olivotto et al., 2001), and congenital heart disease (Hu and Lin, 2019). The lifetime AF risk of 20% for a patient without risk factors rises to 38% in the presence of just one elevated risk factor (Staerk et al., 2018).

In the interim period between the first pharmacological therapy for heart failure-associated AF using the digitalis leaf 234 years ago by William Withering in 1785 (Withering, 1785; Wray et al., 1985) and the explosive growth of catheter ablation therapy for AF in the last decade, several additional antiarrhythmic drugs have been developed. Yet, none has translated into significant gains in AF clinical management. With each new antiarrhythmic drug discovery, hopes for an effective and safe AF panacea rose, but soon ebbed away. However, despite all its limitations during more than two centuries to prevent or permanently cure AF, antiarrhythmic drug therapy has been and will remain a cornerstone of AF clinical management.

### Review Scope

In this primarily pharmacological review, our goal is to examine the benefits (Table 1) and limitations (Table 2) of early, current, and emerging antiarrhythmic drug therapy for AF control. We will also examine the translation gap between the latest mechanistic insights and the presently outdated therapeutic paradigms. We will not acute management of the first AF occurrence or anticoagulant therapy for AF-related stroke prevention. Nor will we discuss antiarrhythmic therapy for lone AF, postoperative AF, AF precipitated by acute precipitating illnesses, or atrial flutter. Instead, we will review some prominent antiarrhythmic drug therapies for recurrent AF. We will focus particularly on the antiarrhythmic drugs featured in selected landmark AF clinical trials that shifted therapeutic paradigms and shaped management guidelines. We will emphasize why we must understand AF natural history to redefine strategies for antiarrhythmic drug development.

**TABLE 1:** Old and current Atrial Fibrillation antiarrhythmic drugs: therapeutic mechanisms and benefits.

Vaughan Williams class	Targets	Therapeutic mechanisms	Therapeutic benefits for Atrial Fibrillation
***Voltage-gated Na*^*+*^*channel blockers***			
IA Quinidine Procainamide Disopyramide	Nav1.5	Open-state I_Na_ block Intermediate offset kinetics ↓ peak I_Na_ → ↓ non-AVN dV/dt_max_, ↑ excitation threshold	Rhythm control ↓ atrial ectopic automaticity ↓ atrial excitability ↓ atrial conduction ↓ Purkinje conduction ↓ reentrant arrhythmias
	K^+^ channels	↓ multiple I_K_’s (I_to_, I_Kr_, I_Ks_, I_K1_, I_KATP_)	↑ atrial ERP ↑ atrial APD
IC Flecainide Propafenone	Nav1.5 SR RyR2-Ca^2+^ channels	Inactivated-state, frequency-dependent I_Na_ block Slow offset kinetics ↓ peak I_Na_ → ↓↓ dV/dt_max_ (non-AVN APs), ↑ excitation threshold ↓ RyR2-mediated SR Ca^2+^ release → ↓ intracellular Ca^2+^ load	Rhythm control ↑ potency at rapid heart rates ↓ atrial ectopic automaticity ↓ atrial excitability ↓ DAD-induced triggered activity Converts unidirectional to bidirectional block → ↓ reentrant arrhythmias ↓ atrial conduction ↓ accessory pathway conduction ↑↑ atrial APD at rapid heart rates
***β-Blockers***			
IIA Non-selective Carvedilol Propranolol Nadolol		↓ G_s_-AC-cAMP signaling → ↓ I_f_ and ↓ I_CaL_ → ↓ RyR2-mediated SR Ca^2+^ release, ↓ intracellular Ca^2+^ load	Rate control ↓ SAN automaticity and ↑ PR ↓ atrial ectopic automaticity ↓ EAD- and DAD-induced triggered activity ↓ AVN conduction ↑ AVN refractoriness ↑ RR ↓ reentrant arrhythmias Prevention of adrenergic AF 1st-line for HFpEF and HFrEF
	Non-selective β-adrenoceptors		
Selective Atenolol Bisoprolol Esmolol Metoprolol	β1-adrenoceptors		
***Na*^*+*^*-K*^*+*^*pump inhibitor***			
IID Digoxin	Muscarinic M_2_ receptors (SAN, atrial, AVN) Na^+^-K^+^ pump	↑ vagal tone ↓ sympathetic tone Hyperpolarizing SAN	Rate control ↓ SAN automaticity ↓ AVN conduction ↑ AVN refractoriness Second-line for HFrEF
***Voltage-gated K*^*+*^*channel blockers***			
III Non-selective Amiodarone Dronedarone	Nonselective K^+^ channels	↓ multiple I_K_’s (I_to_, I_Ks_, I_K1_, I_KACh_)	Rhythm control ↑ APD ↑ atrial ERP ↓ reentrant arrhythmias
Selective Sotalol Dofetilide	Kv11.1 (hERG)	↓ I_Kr_	↓ SAN automaticity (amiodarone) ↓ AVN conduction (amiodarone)
***Voltage-gated Ca^2+^ channel blockers***			
IV Diltiazem Verapamil	Cav1.2 Cav1.3	↓ I_CaL_ → ↓ RyR2-mediated SR Ca^2+^ release, ↓ intracellular Ca^2+^ load	Rate control ↓ SAN automaticity ↑ atrial ERP ↓ AVN conduction ↓ EAD- and DAD-induced triggered activity ↓ reentrant arrhythmias

AC, adenylyl cyclase; AF, atrial fibrillation; AP, action potential; APD, action potential duration; AVN, atrioventricular node; cAMP, cyclic adenosine monophosphate; dV/dt_max_, maximum action-potential upstroke velocity; EAD/DAD, early/delayed afterdepolarizations; ERP, effective refractory period; G_s_, stimulatory G protein; HFpEF (HFrEF), heart failure with preserved (reduced) ejection fraction; RyR2, ryanodine receptor two; SAN, sinoatrial node; SR, sarcoplasmic reticulum.

**TABLE 2:** Old and current Atrial Fibrillation antiarrhythmic drugs: therapeutic mechanisms and benefits.

Vaughan Williams class	Targets	Proarrhythmic mechanisms	Proarrhythmic risks
***Voltage-gated Na*^*+*^*channel blockers***			
IA Quinidine Procainamide Disopyramide	Nav1.5	↓ ventricular I_Na_	No atrial selectivity ↓ ventricular conduction
	K^+^ channels	↓ ventricular I_K_	↑ ventricular APD → ↑ EAD-mediated triggered activity ↑ ventricular ERP ↑ QT → VT/torsades de pointes
	Vagal efferent nerves (SAN, AVN)	Anticholinergic activity (SAN, AVN)	↑ SAN automaticity ↑ AVN conduction → Rapid ventricular response ↑ digoxin toxicity (quinidine)
IC Flecainide Propafenone	Nav1.5 K^+^ channels	Frequency-dependent I_Na_ block ↓ ventricular I_K_	No atrial selectivity ↑ toxicity at rapid heart rates ↓ ventricular conduction ↑ ventricular APD ↑ ventricular QRS ↑ VT/sudden cardiac death (flecainide)
***β-Blockers***			
IIA Non-selective Carvedilol Propranolol Nadolol	Non-selective β-adrenoceptors	↓ AVN/ventricular I_CaL_	No atrial selectivity Bradycardia AV block ↑ vagally-mediated AF ↓ myocardial contractility upregulation of β-adrenoceptors with chronic use β-blocker withdrawal
Selective Atenolol Bisoprolol Esmolol Metoprolol	β1-adrenoceptors		
***Na*^*+*^*-K*^*+*^*pump inhibito*r**			
IID Digoxin	Na^+^-K^+^ pump Muscarinic M_2_ receptors (SAN, atrial, AVN)	↑ sympathetic tone at high doses	No atrial selectivity ↓ atrial ERP (small effect) Narrow therapeutic window ↑ AV block ↑ DAD-mediated triggered activity ↑ VT and arrhythmic death
***Voltage-gated K*^*+*^*channel blockers***			
III Sotalol Dofetilide Amiodarone Dronedarone	K^+^ channels	↓ ventricular I_Kr_	↑ ventricular APD → ↑ EAD-mediated triggered activity ↑ QT → VT/torsades de pointes ↑ QRS Ashman phenomenon
***Voltage-gated Ca*^*2+*^*channel blockers***			
IV Diltiazem Verapamil	Cav1.2 Cav1.3	↓ I_CaL_	No atrial selectivity Bradycardia AV block ↑ rapid ventricular response in AF with WPW syndrome


AF, atrial fibrillation; APD, action potential duration; AV(N), atrioventricular (node); EAD/DAD, early/delayed afterdepolarizations; ERP, effective refractory period; G_s_, stimulatory G protein; I_CaL_, L-type Ca^2+^ current; I_Kr_, rapid delayed rectifier current, SAN, sinoatrial node; VT, ventricular tachycardia; WPW, Wolff-Parkinson-White.

## Early Antiarrhythmic Drugs for Rhythm Control

Historically, the therapeutic approach was a “one-fits-all” paradigm that focused on the antiarrhythmic drugs rather than on the patients’ AF. The classical concept of an ideal pharmacotherapy for AF is an antiarrhythmic drug that can restore and maintain sinus rhythm with minimal or acceptable systemic side effects. In the 20th century, while both rhythm control and rate control were generally acceptable strategies for long-term AF management, the rhythm control strategy, which relies on cardioversion and antiarrhythmic drug therapy, was by far the preferred initial therapy. Classical AF antiarrhythmic drugs most commonly target ion channels for current inhibition rather than current augmentation (Table 1). Unfortunately, because these ion channel blockers commonly lack exquisite target specificity, they block both atrial and ventricular ion channels. As a result, adverse effects, largely from ventricular ion channel blockade (Table 2), often blunt atrial therapeutic benefits (Table 1).

### Class IA Drugs (Quinidine, Procainamide, Disopyramide)

Quinidine, one of the oldest antiarrhythmic drugs, was used extensively in the 20th century for a variety of arrhythmias, including AF (Askey, 1946; McMillan and Welfare, 1947; Wegria, 1947). The combination of quinidine and verapamil has similar efficacy in preventing AF recurrences as sotalol but with better safety profile (Fetsch et al., 2004; Patten et al., 2004).

Procainamide and disopyramide came next on the market as promising new agents for AF in the mid-1900s (McCord and Taguchi, 1951; Miller et al., 1952; Hartel et al., 1974). Classified as Vaughan Williams class IA, quinidine, procainamide, and disopyramide block not only Na^+^ channels, but also K^+^ channels, thereby prolonging effective refractory period (ERP) (Vaughan Williams, 1984). ERP prolongation is beneficial in reducing susceptibility to reentry and therefore AF recurrences.

However, the lack of atrial selectivity of these antiarrhythmics contributes to QRS and QT prolongation, thereby increasing the risk of torsades de pointes, a most serious adverse effect that can lead to sudden cardiac arrest. There is also evidence that class IA drugs, especially disopyramide, decrease vagal tone by inhibiting cholinergic activity, which may require the concomitant use of β-blockers or Ca^2+^ channel blockers (Warrington and Hamer, 1980; Nakajima et al., 1989). For example, thanks to its negative inotropy, disopyramide received class IIa recommendation with level of evidence C for maintenance of sinus rhythm in AF patients with underlying hypertrophic cardiomyopathy, but must be used in conjunction with a β-blocker or nondihydropyridine Ca^2+^ channel blocker (January et al., 2014).

Thus, despite moderate efficacy in preventing AF recurrences, clinical adverse events, especially ventricular proarrhythmia and resultant increased mortality (quinidine and disopyramide), mar the safety profile of these class IA rhythm-control drugs (Lafuente-Lafuente et al., 2015).

## Evolving Therapeutic Strategies

The 21st century marks critical milestones that shifted existing therapeutic paradigms and shaped management guidelines. The first milestone came in 2001, when the ACC, the AHA, and the European Society of Cardiology (ESC) jointly established a unified classification scheme for AF (Fuster et al., 2001; Calkins et al., 2007). Nonsustained recurrent AF is designated “paroxysmal” if AF does not sustain past 7 days. Past 7 days, AF is considered sustained. Sustained AF is designated “persistent” if not lasting longer than 1 year, “longstanding persistent” if lasting longer than 1 year (Calkins et al., 2007; Kirchhof et al., 2016), and “permanent” if rhythm control is unsuccessful or not attempted. Permanent AF is reclassified as longstanding persistent if a rhythm control strategy is adopted after 1 year of continuous AF. By separating AF presentations based on their temporal heterogeneities, this classification scheme marked an important shift in the therapeutic paradigm. The classical “one-fits-all,” drug-centered approach was replaced by the modern arrhythmia-centered realization that no single therapeutic strategy best serves all AF classes. Different AF classes are associated with different prognoses and require different therapeutic strategies, such as rhythm vs. rate control, pharmacological vs. non-pharmacological intervention (Table 3).

**TABLE 3:** Management strategy for recurrent Atrial Fibrillation based on episode duration.

AF classification	Paroxysmal	Persistent^a^	Permanent^a^
AF episode duration	≤7 days	>7 days and ≤1 year	Typically >1 year
Structural remodeling Severity	Atrial fibrosis Minimal to mild	Atrial fibrosis Mild to moderate	Atrial and ventricular fibrosis Moderate to severe
Electrical remodeling Severity	Pulmonary veins Focal	Atria Diffuse	Atria Diffuse
Pathology	Minimal	Atriomyopathy	Cardiomyopathy
Main strategy	Rhythm control	Rhythm or rate control^b^	Rate control only
First-line therapy^b^	Catheter ablation	Antiarrhythmic drugs^c^	Antiarrhythmic drugs^d^
Second-line therapy	Antiarrhythmic drugs^c^	Catheter ablation	N/A

AF, atrial fibrillation.

a Longstanding persistent AF straddles persistent and permanent AF because cardiac remodeling severities and pathology resemble those in permanent AF, but rhythm-control strategy is an option.

b Exceptions: other clinical factors for considerations, such as drug side effect profile, tolerability, and ease of administration or patients’ age, therapeutic preferences, therapeutic contraindications, and comorbidities (hemodynamic status, ongoing myocardial infarction, hypotension, decompensated heart failure, pre-excitation, hypertrophic cardiomyopathy, other structural heart abnormalities unrelated to AF, etc.).

c For rhythm or rate control.

d For rate control only.

The next milestone came from the revolutionary insights of the *Atrial Fibrillation Follow-up Investigation of Rhythm Management* (AFFIRM) (Wyse et al., 2002; Corley et al., 2004), the largest randomized controlled clinical trial at the time (*n* = 4,060 AF patients; 4-years follow up). The goal of the AFFIRM trial was to address the controversy of anticoagulated rhythm control vs. anticoagulated rate control as the main long-term therapeutic strategy with superior survival benefits. The AFFIRM trial revealed that while rhythm control improved survival, the existing antiarrhythmic drugs themselves did not improve survival because adverse effects of the drugs offset any survival benefits by the drugs in maintaining sinus rhythm (Corley et al., 2004). Therefore, antiarrhythmic drug therapies achieved similar outcomes with either the rhythm-control or the rate-control strategy. Nonetheless, this trial contributed in shifting the therapeutic paradigm from prior rhythm control to rate control as the main strategy.

However, improved survival is only one of the two primary clinical outcome endpoints in AF clinical management. The other primary therapeutic outcome is improved quality of life. Following the AFFIRM trial, multiple other randomized controlled trials addressed the question whether rhythm control or rate control was the strategy to superior improvement in quality of life. For example, the *Controlled Study of Rate vs. Rhythm Control in Patients with Chronic AF and Heart Failure* (CAFÉ-II) found that the rhythm-control strategy, even when achieved by an antiarrhythmic drug such as amiodarone, was associated with greater symptomatic relief and improved quality of life as compared to the rate-control strategy (Shelton et al., 2009).

Although an in-depth discussion of non-pharmacological interventions to control AF is beyond the scope of this pharmacological review, a brief comparison of the different merits and shortcomings of pharmacological and non-pharmacological approach, most notably catheter ablation, is warranted.

Overall, non-pharmacological interventions for AF has evolved a long way from electrical cardioversion and surgical ablation to device-based therapy with Holter or pacemaker telemonitoring and catheter ablation. The first non-pharmacological intervention for AF was electrical cardioversion to defibrillate the atria for rhythm control of non-persistent AF or symptomatic management, but its recent use as a diagnostic rather than therapeutic procedure has emerged. To the same end, implantable atrial defibrillator (IAD) for symptomatic, drug-refractory AF was evaluated in a multicenter study (*n* = 136 patients, 40-months median follow-up) but long-term outcome was not encouraging (Geller et al., 2003). Next, instead of atrial defibrillation, permanent atrial pacing to prevent and/or terminate AF has been explored, but there is insufficient evidence to support recommendation for AF therapy unless otherwise indicated for symptomatic bradycardia or sick sinus syndrome (Ellenbogen, 2007).

In the interim, ablation of AF has evolved from the first classical labyrinth Cox-Maze open-heart surgery with cardiopulmonary bypass support in 1987 to less invasive surgical and particularly catheter procedures. Various energy sources (such as cryothermia or ultra-low temperature cryoablation, unipolar or bipolar radiofrequency, microwave, laser, ultrasound, or electroporation) have been experimented to effectively and safely cause atrial myocyte death in a contiguous, transmural, permanent, and linear fashion. The goal of AF ablation is to electrically isolate common sites of triggers that initiate and/or maintain AF. The most common targets for ablation are the pulmonary veins (Haissaguerre et al., 1998) and left atrial appendage (Di Biase et al., 2010; Hocini et al., 2011); less common targets include the left atrial roof, mitral valve isthmus, and atrioventricular junction. Various refinements beyond pulmonary vein isolation, including complex fractionated atrial electrograms (CFAEs), focal impulse and rotor modulation (FIRM), electrocardiographic imaging (ECGI), have been explored but met with variable, inconsistent success (Weiss et al., 2016). For left atrial appendage occlusion, the AtriClip device has gained increasing popularity over the last decade as a safe and efficacious alternative to surgical closure and can be easily deployed during concomitant open chest surgery or stand-alone minimally invasive procedure (Ellis et al., 2017; Caliskan et al., 2018; Toale et al., 2019).

Taken together, while antiarrhythmic drug therapy repeatedly fell short of efficacy and safety expectations, the last 2 decades and particularly the last 5 years mark a revolutionizing milestone in non-pharmacological interventions for rhythm control with tremendous advances in AF catheter ablation (Kuck et al., 2016). For example, the *Catheter Ablation vs. Standard Conventional Therapy in Patients with Left Ventricular Dysfunction and Atrial Fibrillation* randomized clinical trial (CASTLE-AF; *n* = 363 patients, 37.8-months median follow-up) (Marrouche et al., 2018) showed that AF catheter ablation led to reduced overall mortality and heart failure-related hospitalizations. Despite all the controversies that it generated, CASTLE-AF contributed to the most recent clinical guideline revision. In early July 2019, the AHA/ACC/HRS published a focused update containing only one revision for rhythm control maintenance: a new, weak class IIb recommendation with level of evidence B-R for AF catheter ablation in heart failure (January et al., 2019).

However, concerning the two primary endpoints of improved survival and improved quality of life, catheter ablation has not yet proven superior to antiarrhythmic drug therapy as demonstrated by the *Catheter Ablation vs. Antiarrhythmic Drug Therapy for AF* randomized clinical trial (CABANA; *n* = 2,204 patients; 5-years follow up) (Moreno and Zamorano, 2014; Packer et al., 2018). In addition, AF catheter ablation faces its own host of considerable limitations, such as higher costs, limited availabilities of skilled electrophysiologists and experienced centers, procedural risks, and patients’ ablation candidacy, to name just a few. Thus, aside from patients’ own values and preferences for a pharmacological vs. non-pharmacological approach, expert consensus in both the ESC and AHA/ACC//HRS guidelines indicates a stronger class of recommendation for catheter ablation to maintain sinus rhythm in the presence of three critical variables: symptomatic recurrences, AF class (≤80% success rate for *paroxysmal* vs. ≤50% for *persistent and longstanding persistent*) (Kirchhof et al., 2016; Calkins et al., 2018), and heart failure (class of recommendation IIb) (January et al., 2019).

## Current Antiarrhythmic Drugs for Rate Control

Recommendations for antiarrhythmic drug therapy for the ventricular rate-control strategy have not changed (January et al., 2019) since the 2014 AHA/ACC/HRS guideline and are summarized in Table 4 (January et al., 2014). For ventricular rate-control of AF patients with preserved left ventricular ejection fraction (LVEF), β-blockers and nondihydropyridine Ca^2+^-channel blockers (CCBs) remain first-line therapies, but differences between their mechanisms of action and pharmacokinetics lead to differences in therapeutic applications. For AF patients with reduced LVEF, β-blockers are the only first-line rate-control drugs whereas CCBs are contraindicated.

**TABLE 4:** Antiarrhythmic drug recommendations for ventricular rate control since 2014.^a^

Comorbidities	No other CV disease	Hypertension HFpEF	HFrEF	COPD
β-Blocker	First-line	First-line	First-line	First-line
Diltiazem	First-line	First-line	—	First-line
Verapamil	First-line	First-line	—	First-line
Digoxin	—	—	Second-line	—
Amiodarone	Second-line	Second-line	Second-line	—

CV, cardiovascular; HFpEF, heart failure with preserved ejection fraction; HFrEF: heart failure with reduced ejection fraction; COPD: chronic obstructive pulmonary disease.

a Based on the 2014 AHA/ACC/HRS guideline for atrial fibrillation management (January et al., 2014).

### Class IIA Drugs (β-Blockers)

The β-adrenergic system is an important regulator of the L-type Ca^2+^ current (I_CaL_). Cardiac β-adrenoceptors (80% β1, 20% β2) belong to the G protein-coupled receptor superfamily. β-Blockers slows ventricular response in AF by indirect inhibition of I_CaL_ via competitive inhibition with β-agonists for binding to β-adrenoceptors. Binding of β-agonists to β-adrenoceptors activates stimulatory G-proteins (G_s_) that activate adenylyl cyclase to generate cyclic adenosine monophosphate (cAMP), which in turn activates protein kinase A to phosphorylate phospholamban, ryanodine receptors, myofilament proteins, and L-type Ca^2+^ channels, thereby increasing I_CaL._ Binding of β-blockers to β-adrenoceptors prevents β-agonists from binding and activating G_s_-dependent signaling. Because the β-blocker dose-response curve for rate control is relatively flat over the clinically-relevant dose range, once the high dose-range is reached, further dose uptitration produces only minimal gains in additional heart rate reduction but can substantially increase the likelihood of adverse events (hypotension). In that case, another drug class should be added.

### Class IID Drugs (Digoxin)

Digoxin has a direct action on the myocardium and an indirect action on the autonomic nervous system. Digoxin directly blocks the myocardial Na + -K+ pump, leading to increased intracellular Ca^2+^ and slightly improved cardiac contractility. However, more prominent and relevant to AF rate control, digoxin indirectly modulates the electrophysiology of the atria, sinoatrial node, and particularly atrioventricular node by modulating the cardiac autonomic nervous system. In AF patients without heart failure, the indirect neural action of digoxin predominates because it is more robust and occurs at lower therapeutic doses (Watanabe, 1985). This explains why digoxin toxicity in the whole animal results primarily from the indirect neural effect but in the isolated heart from the direct myocardial effect (Levitt et al., 1976b). Of note, in heart failure, because baseline vagal tone is reduced and sympathetic tone is increased, the direct myocardial action of digoxin may predominate (Goodman et al., 1975; Watanabe, 1985).

Interestingly, the mechanisms underlying the pharmacodynamics effects of digoxin on the atrioventricular node caused quite a controversy, but they highlight both the influence of digoxin on the autonomic nervous system and the complex contributions of the autonomic nervous system to AF pathogenesis. Digoxin minimally modulates the electrophysiology of the atria (by reducing atrial refractoriness) and the sinoatrial node (by depressing its activity). In contrast, digoxin exerts profound vagomimetic and small sympatholytic effects on the atrioventricular node, leading to marked increase of atrioventricular nodal refractoriness. This results in reduced impulse conduction to the ventricles and slowing of ventricular response (Goodman et al., 1975; Watanabe, 1985). An important clinical implication is that because the therapeutic mechanism of digoxin depends on intact rich autonomic innervation of the atrioventricular node; its rate-control effect will be attenuated during exercise (that causes withdrawal of vagal tone), blunted in failing hearts, and ineffective in denervated transplanted hearts (Goodman et al., 1975).

Another important clinical implication is that the synergistic or antagonistic interactions of digoxin with other drugs that also act on the autonomic nervous system may potentially lead to disastrous clinical consequences. For example, the potent anticholinergic activity of Class IA antiarrhythmic drugs may antagonize the vagal stimulation by digitalis, potentially leading to deleterious increases in ventricular response. On the other hand, the antiadrenergic activity of β-blockers and other antihypertensive drugs may potentiate the sympathetic inhibition by digoxin, leading to excessively slow ventricular response or even atrioventricular block with resultant fatigue, syncope, or even sudden death. Likewise, the impairment of atrioventricular conduction by Ca^2+^-channel blockers may potentiate that by digoxin, leading to dangerously slow ventricular response (Watanabe, 1985).

A most important digoxin cardiotoxicity is cardiac arrhythmias, typically in the form of atrioventricular block with or without enhanced automaticity. Additionally, although at therapeutic doses the predominant action of digoxin is vagal stimulation, at high doses digoxin action may paradoxically switch from sympathetic inhibition to sympathetic stimulation, potentially causing lethal ventricular tachyarrhythmias (Watanabe, 1985).

### Class IV Drugs (Nondihydropyridine Ca^2+^-Channel Blockers)

I_CaL_ contributes to the upstroke of atrioventricular-node action potentials. Unlike β-blockers, CCBs inhibit I_CaL_ directly by binding to L-type Ca^2+^ channels. Therefore, while both β-blockers and CCBs block I_CaL_, the difference in their mechanisms of action allows synergism. In contrast to the flat β-blocker dose-response curve, the CCB dose-response curve is relatively linear over the clinically relevant dose range. Therefore, CCB dose uptitration produces appreciable gains in additional ventricular rate reduction throughout the full therapeutic range. In a head-to-head comparative clinical trial of emergency patients presenting with AF and rapid ventricular response, diltiazem achieves rate-control targets faster without any increase in adverse events compared to metoprolol (Fromm et al., 2015).

## Current Antiarrhythmic Drugs for Rhythm Control

Recommendations for antiarrhythmic drug therapy for the rhythm control strategy have not changed (January et al., 2019) since the 2014 AHA/ACC/HRS guideline (Table 5) (January et al., 2014).

**TABLE 5:** Antiarrhythmic drug recommendations for rhythm control since 2014.^a^

Comorbidities	No structural heart disease	Coronary artery disease	Heart failure
Flecainide (class IC)^b^ ^,^ ^c^	First-line	—	—
Propafenone (class IC)^b^ ^,^ ^c^	First-line	—	—
Sotalol (class III)^b^ ^,^ ^d^	First-line	First-line	—
Dronedarone (class III)	First-line	First-line	—
Dofetilide (class III)^b^ ^,^ ^d^	First-line	First-line	First-line
Amiodarone (class III)	Second-line	Second-line	First-line

a Based on the 2014 AHA/ACC/HRS guideline for atrial fibrillation management (January et al., 2014).

b Avoid in severe left ventricular hypertrophy (wall thickness > 1.5 cm).

c To combine with atrioventricular nodal blocking drugs.

d Caution in high risk for torsades de pointes.

### Class IC Drugs (Flecainide, Propafenone)

Both flecainide and propafenone are recommended first-line therapy for AF patients with no structural heart disease to restore sinus rhythm acutely or to maintain sinus rhythm long-term (Anderson et al., 1989; Meinertz et al., 2002). These two potent Na^+^ channel blockers slow atrial conduction, thereby reducing atrial automaticity and excitability. Due to their frequency dependence, their potency and, unfortunately, their proarrhythmic toxicity increase with rapid heart rates. Because these drugs lack atrial selectivity, they also act on the ventricular tissue to prolong both action potential and QRS. A QRS prolongation of 25% on therapy over baseline would require dose reduction or drug discontinuation due to the risk of lethal ventricular tachyarrhythmias and hemodynamic collapse. In fact, the contraindication of these drugs in structural heart disease stems from the demonstration of increased mortality associated with flecainide in the *Cardiac Arrhythmia Suppression Trial* (CAST) (Echt et al., 1991; Anderson et al., 1994). Due to similar concern for increased risk of sudden cardiac death, another important contraindication is the congenital loss-of-function Na^+^ channel mutations, such as in Brugada syndrome.

### Class III Drugs (Sotalol, Dofetilide, Amiodarone)

Together with flecainide and propafenone, sotalol is also indicated first-line therapy in the maintenance of sinus rhythm for AF patients with no structural heart disease (Camm et al., 2010; Fuster et al., 2011). The antiarrhythmic benefit for AF and proarrhythmic toxicity of the class III drugs come from their potency in prolonging the action potential and refractoriness of the atria and ventricles, respectively.

Sotalol is particularly potent and proarrhythmic at slower heart rates due to its reverse frequency dependence. Thus, unlike the frequency-dependent class IC antiarrhythmics (flecainide) that are effective in terminating AF, the reverse-frequency dependent class III antiarrhythmics like sotalol and dofetilide are ineffective in terminating AF, but effective in preventing AF. One randomized placebo-controlled clinical trial (*n* = 300 patients with recurrent symptomatic AF; 1-year follow up) found that sotalol and propafenone are equally effective in maintaining sinus rhythm (73 and 63%, respectively), both being superior to placebo (35%) (Bellandi et al., 2001). However, only sotalol caused malignant proarrhythmia (4%) within 72 h of therapy initiation (Bellandi et al., 2001). Due to the lack of atrial selectivity, the class III proarrhythmic toxicity of sotalol relates to its potency in prolonging the QT interval, resulting in its propensity to cause torsades de pointes. The higher the dose and the slower the heart rate, the higher the risk of severe QT prolongation and consequently the higher the risk of torsades de pointes and mortality (Lafuente-Lafuente et al., 2015).

Whereas sotalol has mixed non-cardioselective β-blocking property, dofetilide is a “pure” class III antiarrhythmic, blocking only the voltage-gated K^+^ channels. Dofetilide was approved by United States Food and Drug Administration (FDA) for use in the United States in 1999 for AF patients without structural heart disease and without severely diminished creatinine clearance. Due to reverse-frequency dependence, dofetilide is more effective in maintaining sinus rhythm than in cardioverting AF as demonstrated by the *Symptomatic AF Investigative Research on Dofetilide* (SAFIRE-D) study (Singh et al., 2000). However, as with sotalol, because dofetilide causes dose-dependent QT prolongation, its major proarrhythmic toxicity is torsades de pointes. With incidence of dofetilide-associated torsades de pointes reportedly as high as 4.7%, drug initiation requires inpatient monitoring and certified physicians.

Amiodarone, an iodinated benzofuran derivative, is the most widely used off-label antiarrhythmic for AF due to its superior efficacy in both cardioversion (Khan et al., 2003) and particularly sinus rhythm maintenance (Roy et al., 2000; Wyse et al., 2002; Singh et al., 2005; Freemantle et al., 2011). Amiodarone displays properties of all four Vaughan Williams classes, but is classified as class III due to its major potency in blocking K^+^ channels to prolong cardiac action potential and refractoriness. Therefore, amiodarone also causes QT prolongation, but not torsades de pointes, probably because it also inhibits multiple other ion channels. Although a major cardiac adverse effect of amiodarone is sinus bradycardia, the use of amiodarone is unfortunately limited by a plethora of non-cardiac toxicities, attributed to the progressive accumulation of its damaging metabolite desethylamiodarone in multiple organs including the lungs, thyroid, liver, as well as the central nervous system. Amiodarone also exhibits multiple drug interactions (e.g., with warfarin) due to its inhibition of the cytochrome P450. Therefore, current clinical practice reserves amiodarone for AF with severe left heart failure or for refractory AF when options for other antiarrhythmic drugs have been exhausted.

## Emerging Antiarrhythmic Drugs for Rhythm Control

There have been renewed efforts in the 21st century to discover either new antiarrhythmic agents or new synergistic combinations of existing and new agents to improve both efficacy and safety for AF antiarrhythmic drug therapy (Table 6). To this end, the modern mechanistic prototype for the ideal antiarrhythmic drug to control AF should include two critical specificities: first, specificity for the *atrial tissue* (to avoid ventricular proarrhythmia) and second, specificity for *AF pathology*.

**TABLE 6:** Emergent antiarrhythmic drugs for AF rhythm control: mechanisms and clinical effects.

Drugs	Targets	Mechanisms	Clinical effects
**Ranolazine** *Vaughan Williams* ID	Nav1.5 Kv11.1 (hERG; I_Kr_)	Therapeutic mechanisms Frequency-dependent block of atrial peak and late I_Na_ → ↓dV/dt_max_, ↑ diastolic excitation threshold, ↓ Ca^2+^ overload Reverse frequency-dependent block of atrial I_Kr_ → counteracts Ach-induced ↓atrial APD	Therapeutic benefits for AF Atrial-selective effects of I_Na_ block ↓ EAD-induced triggered activity ↑ atrial ERP ↑ atrial postrepolarization refractoriness Frequency-dependent ↑ atrial conduction time ↓ phase-3 EADs, DADs, triggered activity ↓ AF duration ↓ AF dominant frequency → ↑ likelihood of defibrillation success ↓ Ach-mediated AF ↑ atrial APD
		Proarrhythmic mechanisms ↓ ventricular I_Kr_ (mitigated by ↓ ventricular late I_Na_)	Proarrhythmic risks Slight ↑ QT (without causing torsades de pointes or ↑transmural dispersion of repolarization)
**Vernakalant** **(RSD1235)** *Vaughan Williams* III	Nav1.5 Atrial K^+^ channels Kv1.5 (I_Kur_), I_KACh_, I_to_ Other K^+^ channels Kv11.1 (hERG; I_Kr_)	Therapeutic mechanisms Atrial-predominant I_Na_ block: Open-state, voltage- and frequency-dependent → ↓ atrial dV/dt_max_ Rapid onset/offset kinetics ↓ late I_Na_ (HEK cells) Open-state ↓I_Kur_ (HEK cells) ↓ I_KACh_	Therapeutic benefits for AF Atrial-selective effects ↓ atrial impulse conduction (at rapid heart rates) ↓ atrial excitability Frequency-dependent ↑ atrial ERP ↑ AVN refractoriness ↑ atrial APD Rapid conversion of recent-onset AF
		Proarrhythmic mechanisms ↓ I_Na_ (SAN) ↓ ventricular I_Kr_ (mitigated by ↓ ventricular late I_Na_)	Proarrhythmic risks Sinus bradycardia Slight ↑ QT (without causing torsades de pointes or ↑transmural dispersion of repolarization)
**Niferidil (RG-2)** *Vaughan Williams* III	K_v_1.5 (atrial-specific) Nav1.5	Therapeutic mechanisms ↓ I_Kur_, ↓I_KACh_, ↓atrial I_Kr_ Open-state, voltage- and frequency-dependent I_Na_ block Rapid kinetics	Therapeutic benefits for AF ↑ atrial APD ↑ atrial ERP ↓ recent-onset AF and ↓ persistent AF
		Proarrhythmic mechanisms ↓I_Kss_ and ↓I_Kur_ (murine ventricles)	Proarrhythmic risks ↑ ventricular APD (mice)
**Vanoxerine (GBR-12909)** *DRI*	Kv11.1 (hERG; I_Kr_) Cav1.2 Nav1.5	Therapeutic mechanisms Strongly frequency-dependent I_Kr_ block > I_CaL_ block > frequency-dependent I_Na_ block	Therapeutic benefits for AF High conversion rate for recent-onset AF
		Proarrhythmic mechanisms ↓ventricular I_Kr_	Proarrhythmic risks ↑ QT → VT/torsades de pointes? (Conflicting studies)
**Antazoline** *First-generation antihistamine (H1-receptor blocker)*	Nav1.5? K^+^ channels?	Therapeutic mechanisms ↓ I_Na_? ↓ I_K_?	Therapeutic benefits for AF High conversion rate for PAF ↑ atrial APD ↑↑ atrial ERP ↑ atrial postrepolarization refractoriness ↑ interatrial conduction time
		Proarrhythmic mechanisms	Proarrhythmic risks Bradycardia Non-sustained sinus tachycardia
**XEN-D0103 (S66913)**	Kv1.5 (atrial-specific)	Therapeutic mechanisms ↓ atrial-specific I_Kur_	Therapeutic benefits for AF: none Atrial-selective ion channel blockade ↑ atrial APD No effect on AF burden
		Proarrhythmic mechanisms	Proarrhythmic risks: None
**A293** **Doxapram** *I*_*K2P*_ *inhibitors blockers*	Atrial-specific K2P channels (TASK-1, TASK-3)	Therapeutic mechanisms ↓ atrial-specific leak current I_K2P_	Therapeutic benefits for AF Atrial-selective ion channel blockade ↑ atrial APD ↑ atrial ERP Prevents atrial electrical remodeling
		Proarrhythmic mechanisms	Proarrhythmic risks: None

ACS, acute coronary syndrome; AF, atrial fibrillation; AP, action potential; APD, action potential duration; DRI, Dopamine reuptake inhibitor; dV/dt_max_, maximum action-potential upstroke velocity; EAD/DAD, early/delayed afterdepolarizations ERP, effective refractory period; PAF, paroxysmal atrial fibrillation; SAN, sinoatrial node; VT, ventricular tachycardia.

The atrial specificity of an antiarrhythmic drug can be enhanced by targeting atrial-predominant currents for blockade, particularly those affected by AF electrical remodeling, such as the ultrarapid delayed rectifier K^+^ current (I_Kur_), the acetylcholine-activated inward-rectifier K^+^ current (I_KACh_), the two-pore-domain K^+^ current (I_K2P_), and the small-conductance Ca^2+^-activated K^+^ current (I_SK_). The AF-pathology specificity of an antiarrhythmic drug can be enhanced by exploiting the voltage- and frequency dependence of its channel blockade. Because atrial tissue with active AF is typically depolarized and has high frequency of depolarization, the more the drug blocking potency depends on depolarized voltage and high frequency of depolarization, the greater its specificity for AF pathology.

### New Class I Drug (Ranolazine)

Ranolazine was initially designed as an anti-ischemic, antianginal drug because it blocks both atrial and ventricular late Na^+^ current (late I_Na_), which increases pathologically during myocardial ischemia. Increased late I_Na_ is proarrhythmic because the resulting intracellular Na^+^ overload activates Na^+^-Ca^2+^ exchanger (NCX) reverse-mode, causing intracellular Ca^2+^ overload that leads to sarcoplasmic reticulum Ca^2+^ leak, diastolic dysfunction, and increased oxygen demand (Hale et al., 2006; Hale et al., 2008). However, ranolazine anti-AF efficacy in AF may also be due to its reduction of peak I_Na_ and especially its atrial-selective inhibition of the rapid delayed rectifier K^+^ current (I_Kr_) carried by Kv11.1, the cardiac human *ether-a-go-go*-related gene (hERG) K^+^ channel. Together, these three mechanisms allow ranolazine to increase atrial postrepolarization refractoriness and slow interatrial conduction without affecting atrial action potential duration (Burashnikov et al., 2007; Kumar et al., 2009; Antzelevitch et al., 2011).

Ranolazine plus amiodarone is one particular combination of antiarrhythmic drug therapy that has shown promising results for synergistic efficacy for conversion of post-cardiac surgery and other recent-onset AF (Koskinas et al., 2014; Simopoulos et al., 2018). For new-onset AF, the combination of ranolazine and amiodarone had higher efficacy in terminating AF than amiodarone alone (Koskinas et al., 2014).Used alone, ranolazine decreased AF recurrence after electrical cardioversion (White and Nguyen, 2017) and reduced the frequency of new onset AF as well recurrences following coronary artery bypass grafting surgery (De Vecchis et al., 2018).

Likewise, the combination of ranolazine and low-dose dronedarone reduced the AF burden (mean duration of AF episodes) by 59% as compared to placebo in the HARMONY clinical trial (Reiffel et al., 2015). The synergistic activity between ranolazine and dronedarone can potentially reduce the dose needed and reduce the adverse effects associated with dronedarone monotherapy.

### New Class III Drugs (Dronedarone, Vernakalant, Niferidil)

#### Dronedarone

Dronedarone is a newer, non-iodinated benzofuran derivative of amiodarone, designed to reduce amiodarone-related non-cardiac toxicities. Iodine moieties were removed to reduce thyroid toxicity. A methylsulfonamide group was added to reduce lipophilicity and consequent accumulation in lipid-rich organs (Zareba, 2006). Disappointingly, comparing with amiodarone, dronedarone efficacy is inferior (Le Heuzey et al., 2010; Freemantle et al., 2011) and its safety profile is different, but not necessarily superior (Hohnloser et al., 2009; Connolly et al., 2011). The most concerning dronedarone non-cardiac toxicity, which received FDA warning, is severe liver injury with rare risk of acute failure requiring transplant.

#### Vernakalant (RSD1235)

Another notable contender is vernakalant (RSD1235), a new class III antiarrhythmic drug with frequency- and voltage-dependent blockade. Originally developed for Kv1.5 (I_Kur_) block to treat AF, vernakalant was shown experimentally to block multiple ion channels, including those mediating I_Kur_, I_KACh_, the transient outward K^+^ current (I_to_), I_Kr_, peak and late I_Na_. Interestingly, vernakalant acts as an atrial-selective antiarrhythmic drug but is not an atrial-selective ion channel blocker. At least two reasons account for its atrial selectivity. First, because the resting membrane potential is more depolarized in the atria than ventricles, plus this voltage difference is further amplified during AF, the dependence of its potency on depolarized membrane voltage confers atrial selectivity. Second, because Kv1.5 channels are detected only in human atria, but not in human ventricles, the blockade of human Kv1.5 by vernakalant is atrial-specific. Aside from its atrial selectivity, its rapid onset and offset kinetics confers a low proarrhythmic risk. Of note, while the Kv1.5 block by vernakalant was demonstrated in Kv1.5-expressing HEK293 cells (Eldstrom et al., 2007) and a rat model of myocardial infarction (Fedida et al., 2005), there has been no evidence yet for Kv1.5 block by vernakalant in human atrial myocytes (Wettwer et al., 2013). Likewise, to date the block of I_NaL_ by vernakalant was demonstrated only in Nav1.5-expressing HEK293 cells and rabbit Purkinje fibers (Fedida et al., 2006; Orth et al., 2006), but not in human atrial tissue (Wettwer et al., 2013).

Instead, the clinical efficacy of vernakalant in acute rhythm control of recent-onset AF is largely attributed to its frequency- and voltage-dependent open-state blockade of Na^+^ channels. Nav1.5 block by vernakalant reduces maximum action-potential upstroke velocity (dV/dt_max_), thereby impairing atrial impulse conduction (Roy et al., 2008; Kowey et al., 2009; Camm et al., 2011). Additionally, its rapid unbinding kinetics of Nav1.5 block by vernakalant contributes to augmenting atrial selectivity and lowering ventricular proarrhythmic risks (Burashnikov and Antzelevitch, 2009).

Multiple placebo-controlled phase 3 studies have demonstrated the superior efficacy of vernakalant in rapid AF cardioversion (8–14 min after the first dose) (Roy et al., 2008; Kowey et al., 2009; Pratt et al., 2010; Stiell et al., 2010; Beatch et al., 2017), particularly in comparison to other antiarrhythmic drugs, such as amiodarone (Camm et al., 2011), propafenone (Conde et al., 2013a), flecainide (Conde et al., 2013b), and ibutilide (Simon et al., 2017).

In Europe, per the 2012 ESC Guidelines on AF management, vernakalant received class I recommendation with level of evidence A for cardioversion of AF with structurally normal heart or minimal heart disease and class IIb recommendation with level of evidence B for cardioversion of patients with moderate structural heart disease (Camm et al., 2012). Unfortunately, despite its high conversion rate for recent-onset AF that would allow rapid and efficacious treatment in the emergency department to reduce cost of care, vernakalant has not been approved by the United States FDA due to lingering safety concerns, including hypotension, bradycardia (a common adverse event), and the increased risk of serious adverse events (≤2%) with usage in heart failure or post-acute coronary syndrome (Lindsay, 2011). The risk of vernakalant inducing sustained ventricular arrhythmias or torsades de pointes is low but may increase with concomitant use of Class I/III antiarrhythmic drugs (Beatch and Mangal, 2016; Akel and Lafferty, 2018). The opposing conclusions regarding vernakalant usage by the regulatory bodies from the two sides of the Atlantic is puzzling considering their examination of the same clinical evidence. Because of the United States’ dominant position in the global marketplace, the FDA decision to withhold approval for vernakalant led sponsors of vernakalant to abandon further refinement pursuits for the drug.

#### Niferidil (RG-2)

Niferidil (RG-2) is a new class III antiarrhythmic drug, first introduced for human use in early 2010s. In patients, niferidil is highly effective for both persistent AF (85% conversion rate within 24 h) and recent-onset AF of less than 3-months duration (92% conversion rate) (Maykov et al., 2014). Because the study lacked randomization and placebo controls, more robust clinical trials are needed to demonstrate niferidil promising efficacy and safety profile in cardioversion of persistent AF. The ionic mechanisms responsible for its unique antiarrhythmic efficacy in human persistent AF have not been investigated. However, in guinea pigs, niferidil prolongs atrial action potential duration via I_Kr_ inhibition (Abramochkin et al., 2017). However, as demonstrated in mice, niferidil has off-target effects in the ventricular myocardium, where it also prolongs ventricular action potential duration in mice by inhibiting two other delayed rectifier K^+^ current, I_Kur_ and the steady-state current (I_ss_) (Abramochkin et al., 2015). It is unclear how probable off-target inhibition of human ventricular K^+^ currents by niferidil affects its efficacy and safety profile as an antiarrhythmic drug for AF. Therefore, minimizing off-target effects to improve efficacy and safety is the motivation to improve atrial selectivity of new antiarrhythmic drugs for AF control.

### New Atrial-Selective Atrial Fibrillation Antiarrhythmic Drugs (Vanoxerine, Antazoline, XEN-D0103, I_K2P_ Blockers)

The need for atrial selectivity galvanized efforts to repurpose existent drugs (vanoxerine, antazoline) and to develop novel antiarrhythmic drugs (XEN-D0103, I_K2P_ blockers) (Table 6).

#### Vanoxerine (GBR-12909)

Vanoxerine is a 1,4-dialkylpiperazine derivative, a potent and selective dopamine reuptake inhibitor originally designed to treat Parkinson’s disease and depression due to its potent dopamine reuptake inhibition. Vanorexine is a potent blocker of I_Kr_, I_CaL_, and I_Na_ in decreased order of potency. I_CaL_ and I_Na_ blocks are strongly frequency-dependent and can offset I_Kr_ block, such that vanorexine does not prolong ventricular action potential duration or the QT interval, nor increase of the transmural dispersion of ventricular repolarization (Lacerda et al., 2010). These mechanisms likely explain why, unlike other class III antiarrhythmic drugs, vanorexine does not cause ventricular arrhythmias in animals (Matsumoto et al., 2010). Therefore, vanorexine resembles amiodarone in therapeutic benefits with its multichannel block and repolarization uniformity, but thankfully not in amiodarone toxicity (Lacerda et al., 2010).

Subsequently, a randomized, double-blinded study confirmed vanoxerine superior efficacy to placebo (70% vs. 20%). However, the study was terminated early due to the occurrence of ventricular arrhythmias and torsades de pointes in the treatment group (Piccini et al., 2016). This concerning finding regarding vanorexine safety profile was in contrast to an earlier phase 2B clinical trial, the *Safety and Efficacy of Vanoxerine for Conversion of Atrial Fibrillation or Flutter to Normal Sinus Rhythm* (COR-ART) trial, which found vanoxerine to have high conversion rate (84%), to be well tolerated, and to cause no ventricular arrhythmias (Dittrich et al., 2015). Clearly, larger studies are required to study the safety and efficacy of vanoxerine before definitive conclusions can be drawn. This may be a worthy endeavor if vanoxerine becomes a viable option for a pill-in-the-pocket approach for paroxysmal AF in structural heart disease where standard antiarrhythmic drugs are contraindicated.

#### Antazoline

Antazoline is a first-generation antihistamine with known antiarrhythmic properties and quinidine-like activities, first reported back in 1960s. Early data of the use of intravenous antazoline in AF cardioversion showed disappointing minimal efficacy (Gehring and Kehler, 1970). Since then, studies on antazoline have been sparse until recent renewed interest. Antazoline has been shown experimentally to reduce AF inducibility by increasing atrial APD, atrial ERP, atrial postrepolarization refractoriness, and interatrial conduction time. Inhibition of several ion currents, including both Na^+^ and K^+^ currents, presumably accounts for its antiarrhythmic effects (Frommeyer et al., 2017).

In the *Efficacy and Safety of Antazoline in the Rapid Cardioversion of Paroxysmal Atrial Fibrillation* (AnPAF) randomized clinical trial (*n* = 74 patients with paroxysmal AF) that antazoline could rapidly restore sinus rhythm (72.2% efficacy in the treatment group vs. 10.5% in the placebo group) and its only serious proarrhythmic effect was non-sustained sinus tachycardia in 5.6% of the treatment group (Maciag et al., 2017). A subsequent retrospective review of medical records from 450 Polish patients with short-duration AF, the *Cardioversion with Antazoline Mesylate* (CANT) study, demonstrated that antazoline represents an efficacious and safe method of pharmacological cardioversion with superior success rate compared to amiodarone and propafenone (Wybraniec et al., 2018). Thus, antazoline has emerged as a promising antiarrhythmic drug with cardioversion efficacy potentially surpassing amiodarone. However, additional randomized clinical trials are necessary to confirm this finding and to investigate its safety profile.

#### XEN-D0103/S66913

In the pipeline is XEN-D0103 (also known as S66913), a novel atrial-selective inhibitor of I_Kur_ carried by the Kv1.5 channel, which expresses only in atria (Ford et al., 2016). A phase one study demonstrated that XEN-D-0103/S66913 may have promising therapeutic benefit for reducing AF burden because it prolonged the atrial action potential duration specifically without affecting the ventricles (Ford et al., 2016). However, a recent prospective single-center phase two double-blind, randomized, placebo-controlled, cross-over study (*n* = 21 patients with paroxysmal AF and implanted dual-chamber permanent pacemaker for conduction defects) found that although XEN-D0103 was well-tolerated, it did not reduce AF burden when used alone (Shunmugam et al., 2018).

A most recent randomized, double-blind, placebo-controlled trial, the *Double-blind, International study AssessinG efficacy of S 066913 in paRoxysmal Atrial Fibrillation*-IKur inhibitor (DIAGRAF-IKUR; *n* = 58 patients with paroxysmal AF being considered for ablation), confirmed prior findings that S66913 was safe but ineffective in reducing AF burden (Camm et al., 2019). However, due to the small sample sizes of these studies, further trials in larger patient samples are still needed to assess its efficacy in cardioversion and maintenance.

#### I_K2P_ Blockers (A293, Doxapram)

Another attractive target in the pipeline are atrial-selective inhibitors of I_K2P_, a K^+^ leak current carried by the most recently discovered K2P channels (Goldstein et al., 2001). Like other leak currents, I_K2P_ contributes to the resting membrane potential and controls myocyte excitability by shaping action potential amplitude, duration, and frequency. Several classic antiarrhythmic drugs, such as amiodarone, dronedarone, carvedilol, are non-selective I_K2P_ blockers; hence, the pressing need to develop selective I_K2P_ blockers for similar efficacy but improved safety.

The Two-pore-domain Weak Inward rectifying K^+^ channel (TWIK)-related Acid-*S*ensitive K^+^ channel-1 (TASK-1; K_2P_3.1) is a member of the K2P channel family (Enyedi and Czirják, 2010). Because TASK-1 channels are abundant in human atria but not in ventricles (Heijman et al., 2017; Schmidt et al., 2017), TASK-1 channels regulate atrial action potential duration (Limberg et al., 2011). In chronic AF, but not in paroxysmal AF, atrial TASK-1-mediated K_2P_3.1 current expression is upregulated and causes action potential shortening (Schmidt et al., 2015). In a preclinical trial with an AF pig model, inhibition of atrial-specific K_2P_3.1 current using the TASK-1 channel blocker A293 reverses pathologic AF-related atrial action potential shortening and significantly prolongs atrial ERP without causing ventricular arrhythmias (Wiedmann et al., 2020). In another more recent pig preclinical trial, K_2P_3.1 current inhibition by genetic ablation of TASK-1 channels using small interfering RNA (siRNA) prevents atrial electrical remodeling and suppresses AF (Schmidt et al., 2019). However, an important caveat is that in chronic-AF patients with heart failure due to left ventricular dysfunction, atrial K_2P_3.1 expression is downregulated instead of upregulated, making it unlikely for I_K2P_ blockers to be an efficient anti-AF approach in patients with structurally remodeled atria (Schmidt et al., 2017).

These encouraging preclinical findings highlight the need to further assess in clinical trials the potential of atrial-selective inhibition of I_K2P_ as a novel, promising mechanism-based strategy to treat AF. In fact, an ongoing clinical trial in Germany, the DOxapram Conversion TO Sinus rhythm study (DOCTOS Trial, EudraCT no. 2018-002979-17, *n* = 40 AF patients aged 18–64 years and *n* = 40 AF patients aged ≥65 years), is evaluating the efficacy and safety of doxapram, a potent atrial-selective I_K2P_ blocker, in cardioverting paroxysmal and persistent non-valvular AF to sinus rhythm within 6 h of intravenous administration. Doxapram, a known ventilator stimulant, blocks both TASK-1 and TASK-3 (K_2P_9.1) channels. TASK-3, the closest relative of TASK-1, expresses prominently in human right auricles. Interestingly, human atrial TASK-1 and TASK-3 can form heterodimers (Rinné et al., 2015).

## Redefining Natural History and Pathophysiological Mechanisms

### Paradigm Shift: From Three Distinct Atrial Fibrillation Temporal Patterns to One Progressive Fibro-Inflammatory “Atriomyopathy”

#### Electrical Remodeling

An early breakthrough in AF pathophysiology came with the recognition of the pathogenic electrical remodeling process. In electrical remodeling, “AF begets AF”—that is AF leads to electrical remodeling of the atrial electrophysiology to facilitate further AF induction and maintenance (Wijffels et al., 1995)—such that by the time AF progresses to the permanent stage, attempts to restore sinus rhythm are doomed to fail and therefore should not be attempted. The main perturbations in AF electrical remodeling that lead to shortening of the atrial action potential duration and resultant shortening of atrial refractoriness include downregulation of I_CaL_ (Yue et al., 1997; Van Wagoner et al., 1999), upregulation of the inward rectifier current (I_K1_) (Biliczki et al., 2019), upregulation of I_SK_ (Qi et al., 2014), and constitutive activation of I_KACh_ (Dobrev et al., 2001; Dobrev et al., 2005; Heijman et al., 2018). The main perturbations in AF electrical remodeling leading to aberrant Ca^2+^ handling and resultant increased likelihood of reentry (Liu et al., 2020) and delayed afterdepolarization (a well-known trigger of AF) include persistent diastolic ryanodine receptor (RyR2) leak due to both sustained sarcoplasmic reticulum Ca^2+^ overloading, Ca^2+^/calmodulin kinase type II (CaMKII) activation, and upregulation of NCX (Nattel and Dobrev, 2012).

#### Structural Remodeling

The next breakthrough in AF pathophysiology came with the recognition of the pathogenic structural remodeling process. Fibrosis and inflammation were recognized as the hallmark of atrial structural remodeling in AF (Nattel, 2016; Yao et al., 2018). The knowledge that fibrosis and inflammation in AF correspond with disease severity and have prognostic value came with the technological advent of catheter ablation interventions for AF and late-gadolinium enhancement magnetic resonance imaging (LGE-MRI) to detect atrial fibrosis *in vivo*. LGE-MRI revealed that atrial fibrosis severity determines AF ablation outcome, likelihood of AF recurrence, stroke risk, and significant sinus node dysfunction; therefore, assessment of atrial fibrosis can guide patient selection and ablation procedures (Akoum and Marrouche, 2014; McGann et al., 2014). Extensive atrial structural remodeling by fibrosis (≥30% LA wall enhancement) predicts poor response to catheter ablation therapy for AF (Marrouche et al., 2014; McGann et al., 2014). Importantly, even when ablation succeeds in restoring sinus rhythm, ablation causes additional inflammation and fibrosis that perpetuate the original electrical and structural remodeling caused by AF (Teh et al., 2012). After AF ablation, the atrial conduction slowing, the decrease in left atrial ejection fraction with or without atrial dilation, and the development of new ablation-induced inflammation and fibrosis (Okumura et al., 2011) indicate worsening atrial function and predict increased risk of AF recurrence (Marrouche et al., 2014; Kim et al., 2018; Kim et al., 2019). Marked atrial fibrosis and elevated levels of inflammatory markers in AF recurrences are evident both *in vivo* (Qu et al., 2009; Sonmez et al., 2014) and postmortem (Ito et al., 2013; Corradi et al., 2014). Collagen volume, activity of nuclear factor κ-light-chain-enhancer of activated B cells (NF-κB, a crucial inflammatory signaling transcription factor) (Qu et al., 2009) and serum levels of fibro-inflammatory biomarkers are all elevated in persistent-AF (Sonmez et al., 2014) and post-ablation AF recurrence (Okumura et al., 2011). Importantly, this elevated fibro-inflammatory state positively correlates with left atrial dilatation, an index of atrial structural (Sonmez et al., 2014).

#### Natural History

A breakthrough in AF natural history came with the realization of the prognostic value of the AF burden (duration and frequency of AF episodes) on disease progression. The knowledge of this longitudinal evolution of AF prompted the American College of Cardiology (ACC), American Heart Association (AHA), and ESC to classify AF based on temporal rhythm patterns into paroxysmal, persistent, and permanent and to recommend indications for therapeutic interventions based on AF class (Fuster et al., 2006; Jahangir et al., 2007; Ogawa et al., 2018).

#### Progressive “Atriomyopathy”

A most significant breakthrough in AF natural history and pathophysiology came with the appreciation that atrial electrical remodeling is intertwined with atrial structural remodeling (Li et al., 1999; Nattel et al., 2008) in a regenerative positive feedback loop that amplifies the pathophysiology of the initial AF episodes. We coined the term “atriomyopathy” to describe the ultimate consequence of the synergy of these two relentless AF pathogenic remodeling processes for the atrial myocardium. The progressive nature of AF atriomyopathy implies a critical need for early rhythm-control therapy in AF because timing matters in preventing or delaying the progression of AF atriomyopathy. This paradigm-shifting therapeutic strategy is most recently validated by the *Early Treatment of Atrial Fibrillation for Stroke Prevention Trial* (EAST-AFNET 4), an international, randomized, open, blinded-outcome-assessment clinical trial (Kirchhof et al., 2020). In fact, the most recently published 2020 AF guidelines mark a revolutionizing paradigm shift in management strategies because the pressing need for primary prevention to thwart AF atriomyopathy onset is emphasized for the first time (Hindricks et al., 2020).

## Redefining Therapeutic Goals

### Paradigm Shift: The Need for a Mechanism-Based Multipronged Strategy

**FIGURE 1:**
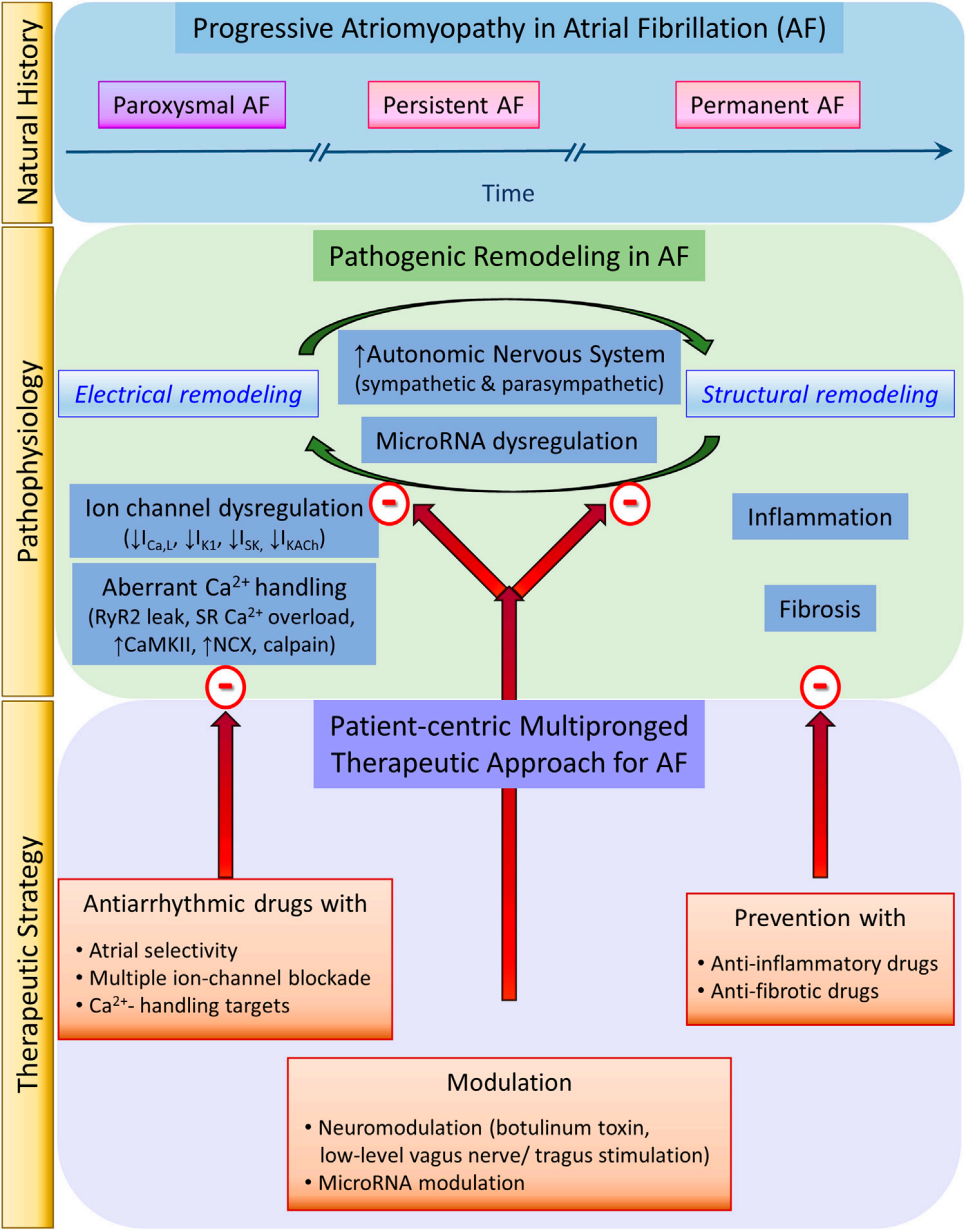
Atrial fibrillation natural history, pathophysiology, and therapeutic strategy.

To date, AF has been treated for rate control by antiarrhythmic drugs and for rhythm control by either antiarrhythmic drugs or invasive ablation. While the invasive Cox-maze and catheter ablation have evolved over the last three-and-a-half and 2 decades, respectively, antiarrhythmic drugs for AF have evolved over three-and-a-half centuries. Yet, our expectations for an efficacious and safe AF management strategy have not been met. Over that same time, thanks to basic and clinical research, our understanding of AF pathophysiology has also evolved from the assumption of AF as a static disease to the appreciation of AF as a progressive fibro-inflammatory atriomyopathy driven by a relentless positive feedback loop between electrical and structural remodeling. Therefore, our AF management focus also needs to evolve from a pure curative intent to incorporate preventive intent. Importantly, our AF management strategy also needs to evolve from the historical “one-fits-all” approach to a modern mechanism-based multipronged strategy to target both pathogenic electrical and structural remodeling processes (Figure 1).

#### Targeting Electrical Remodeling

To prevent or forestall pathogenic electrical remodeling with efficacy and safety, basic research has revealed at least three novel aspects to focus the development of antiarrhythmic drugs: atrial selectivity, multiple-ion-channel blockade, and Ca^2+^-mishandling targets. Novel potential ionic current targets include the currents carried by K^+^ channels with atrial-predominant expression, such as I_Kur_, I_K2P_, I_SK_, and I_KACh_. Ca^2+^ mishandling also offers promising targets for stabilizers of RyR2, modulators of the sarcoplasmic reticulum Ca^2+^-ATPase 2a (SERCA2a), and inhibitors of NCX, CaMKII, and Ca^2+^-dependent protease calpain.

#### Targeting Structural Remodeling

Unlike electrical remodeling, which offers opportunities for reversibility, to date structural remodeling has not been meaningfully reversed once established. Therefore, prevention is the best approach to target pathogenic structural remodeling (Benjamin et al., 2009; Savelieva et al., 2011a; Savelieva et al., 2011b). Clinical targeting of modifiable AF risk factors such as diabetes, hypertension, obesity, and obstructive sleep apnea should be early and aggressive. Statins, and anti-inflammatory drugs targeting cytokines (IL-1β, IL-6, and TNF-α) and the renin-angiotensin-aldosterone system (RAAS) have been tried as potential therapies to forestall the progression of inflammation and fibrosis (Savelieva et al., 2011a; Savelieva et al., 2011b; Chaugai et al., 2016). Unfortunately, clinical success is still lacking, consistent with the irreversibility of structural remodeling.

#### Targeting Both Electrical and Structural Remodeling

Other promising areas of active research in modulation of microRNA (miRNA) expression and of the autonomic nervous system focus on mechanisms that target both electrical and structural remodeling simultaneously. Basic and clinical science have recently discovered the central signaling role of miRNAs in AF pathogenesis (Luo et al., 2015; Shen et al., 2020). Multiple dysregulated miRNAs, including miR-1, miR-26, miR-106b-25, miR-208a, miR-328, and miR-499, contribute to AF electrical remodeling (Lu et al., 2010; Ling et al., 2013; Luo et al., 2013; Soeki et al., 2016). These miRNAs target and dysregulate the expression of genes encoding critical atrial K^+^ and Ca^2+^ ion channels to cause shortening atrial action potential duration and effective refractory period to favor reentry for AF maintenance. Other multiple dysregulated miRNAs, including miR-21, miR-26, miR-29, miR-30, miR-133, and miR-590, contribute to AF pro-fibrotic structural remodeling (Dawson et al., 2013; Cheng et al., 2019; Sieweke et al., 2020). Therefore, modulating miRNAs has the potential of an innovative AF management strategy of important therapeutic, diagnostic, and prognostic values.

Neural modulation promises another frontier in AF rate and rhythm control given the prominent role of neural remodeling in AF initiation and maintenance (Chen et al., 2014). Autonomic nervous system activation of either limb or both limbs simultaneously can promote AF (Coumel, 1993). Neural remodeling perpetuates AF by engaging a positive feedback loop with adverse atrial electrical and structural remodeling. Sympathetic stimulation induces adrenergic AF (Lu et al., 2008) and facilitates cholinergic AF induction (Hashimoto et al., 1968; Sharifov et al., 2004). Sympathetic stimulation induces adverse atrial neural remodeling by heterogeneous sympathetic hyperinnervation and extensive nerve sprouting (Jayachandran et al., 2000; Chang et al., 2001). Sympathetic stimulation induces adverse atrial electrical remodeling by causing cellular Ca^2+^ overload and mishandling, activating NCX forward-mode and CaMKII, thereby promoting the emergence of AF triggers and shifting of pacemaker activity to ultrarapid-firing ectopic atrial sites (Geesbreght and Randall, 1971). Sympathetic stimulation induces adverse structural remodeling by causing myocyte apoptosis and myocardial fibrosis. Hence, to prevent or reverse AF electrical and structural remodeling, suppression of sympathetic outflow has been investigated using antiadrenergic drugs, renal sympathetic denervation (Wang et al., 2013), or ablation of atrial ganglionated plexi. Sympathetic suppression may reduce AF burden and inducibility and improve the success of pulmonary vein isolation (Katritsis et al., 2011), but success has proved inconsistent.

More curious are the paradoxical contributions of vagal stimulation to both AF pathogenesis and AF therapy (Chen et al., 2014). Vagal stimulation induces cholinergic AF (Hashimoto et al., 1968; Levitt et al., 1976a), also by causing adverse electrical and structural remodeling. Vagal stimulation induces adverse atrial electrical remodeling by heterogeneous shortening of atrial ERP (Liu and Nattel, 1997) and increasing dispersion of atrial refractoriness (Alessi et al., 1958), heterogeneous shortening of atrial action potential duration and increasing dispersion of atrial repolarization as well as increasing interatrial dyssynchrony (Ninomiya, 1966). Vagal stimulation induces profibrotic cardiac structural remodeling. For example, following T_5_ spinal cord transection, parasympathetic hyperinnervation causes dilation of left ventricular chamber, thinning of myocardial wall, and increased collagen content (Lujan et al., 2014). Therefore, it comes as no surprise that suppression of vagal stimulation by anticholinergic drugs or vagal denervation may improve the efficacy of pulmonary vein isolation to prevent AF recurrence (Chen and Tan, 2007) and reduces AF inducibility (Elvan et al., 1995). Likewise, suppression of vagal stimulation by ablation of pulmonary vein ganglionated plexi may increase the success of pulmonary vein isolation by mitigating vagally-induced shortening of atrial ERP (Lemola et al., 2008). Interestingly, botulinum toxin, known to inhibit vesicular release of intracellular acetylcholine from presynaptic neurons, presents a novel neuromodulation approach in AF therapeutics by simultaneous sympathovagal suppression. Recently, two prospective, double-blind study of patients with paroxysmal AF undergoing coronary artery bypass grafting, found that botulinum toxin injected into the epicardial fat pad suppressed AF both early post-operatively and, surprisingly, long-term during 1-year (Pokushalov et al., 2015) and 3-years follow up (Romanov et al., 2019). The unexpected salutary long-term antiarrhythmic effects of botulinum toxin were attributed to its suppression of the ganglionated plexi activity (Pokushalov et al., 2015).

Thus, it may seem shockingly heretical at first to suggest vagal stimulation as a promising candidate for the new frontier in AF therapeutics. Yet, it has long been recognized that digoxin controls ventricular response during AF by vagal stimulation of the atrioventricular node. Likewise, direct electrical stimulation of the right inferior ganglionated plexus may slow ventricular response in postoperative AF by slowing atrioventricular node conduction. However, the emerging paradigm of AF neuromodulation by vagal stimulation relies on another mechanism to suppress AF, namely by preventing or reversing atrial electrical and structural remodeling. Not all vagal stimulation is arrhythmogenic; the proarrhythmic vs. antiarrhythmic effects are dictated by the strength and duration of vagal stimulation and the complex but poorly understood interactions between the extrinsic and intrinsic cardiac autonomic nervous system. For example, in a dog model of tachypacing-induced AF, strong vagal nerve stimulation that slows the sinus rate by at least 60% facilitates AF inducibility, but moderate vagal nerve stimulation that slows the sinus rate by only 10–30% does not shorten atrial ERP or affect AF inducibility (Takei et al., 2001; Zhang et al., 2009a). Low-level stimulation of the extrinsic cardiac autonomic nervous system can suppress AF by inhibiting the autonomic outflow of intrinsic cardiac autonomic nervous system (Zhang et al., 2009c; Shen et al., 2011; Yu et al., 2011). In fact, low-level vagus nerve stimulation (LLVS) can suppress tachypacing-induced adrenergic AF as well was cholinergic-induced AF by mitigating neural remodeling by the intrinsic cardiac autonomic nervous system, thereby preventing and reversing adverse atrial electrical and structural remodeling (Sheng et al., 2011; Kusunose et al., 2014; Beaumont et al., 2015). LLVS suppresses post-operative AF and attenuates inflammation (decreased serum TNF-α and IL-6) (Zhang et al., 2009b; Rossi et al., 2012; Stavrakis et al., 2017). Likewise, in dogs and humans, transcutaneous low-level tragus electrical stimulation (LLTS) suppresses AF and attendant generation of inflammatory cytokines in patients with paroxysmal AF (Stavrakis et al., 2015). The *Transcutaneous Electrical Vagus Nerve Stimulation to Suppress Atrial Fibrillation* (TREAT-AF), a sham-controlled, double-blind, randomized clinical trial just completed in April 2020, found that intermittent LLTS lowers the AF burden in patients with paroxysmal AF compared to sham control stimulation (Stavrakis et al., 2020).

From these exciting experimental and clinical findings regarding neuromodulation as AF treatment, we learn at least two important lessons. First, to improve efficacy and safety, future antiarrhythmic drugs should have not only atrial selectivity but also dual adrenergic and cholinergic activities (like amiodarone) instead of only a single activity. Second, discrepancies in findings and inconsistencies in success surrounding the use of neuromodulation for AF control highlight the complexity of the dynamic influences of the cardiac autonomic nervous system on AF pathogenesis and the need for future research to guide appropriate identification of patients who would most benefit from neuromodulation for AF prevention or AF control based on individual variability in autonomic influences on existing cardiac structural and electrical remodeling.

### Paradigm Shift: From Drug-Centricity to Patient-Centricity

In summary, as the progressive nature of AF atriomyopathy and the synergy of the two pathogenic remodeling processes in AF becomes clearer, identifying novel therapeutic targets and strategies will progress. We may also become better at considering individual variabilities in genes, environment, and lifestyle to bring precision medicine to AF therapeutics. The hope is to shift the overarching AF therapeutic paradigm from drug-centricity to patient-centricity.

## Author Contributions

MG and AL performed literature search and assisted with manuscript preparation. TN conceived the review, performed literature search, and prepared the manuscript. All authors contributed to manuscript revision, approved the submitted version, and agreed to be accountable for all aspects of the work.

## Funding

This work was supported by the National Institutes of Health R01 HL141452 to TN.

## Conflict of Interest

The authors declare that the research was conducted in the absence of any commercial or financial relationships that could be construed as a potential conflict of interest.

## Clinical Trial Acronyms

AFFIRM, Atrial Fibrillation Follow-up Investigation of Rhythm Management; AnPAF, Efficacy and Safety of Antazoline in the Rapid Cardioversion of Paroxysmal Atrial Fibrillation; CABANA, Catheter Ablation vs. Antiarrhythmic Drug Therapy for AF; CAFÉ-II, Controlled Study of Rate vs. Rhythm Control in Patients with Chronic AF and Heart Failure; CANT, Cardioversion with Antazoline Mesylate; CAST, Cardiac Arrhythmia Suppression Trial; CASTLE-AF, Catheter Ablation vs. Standard Conventional Therapy in Patients with Left Ventricular Dysfunction and Atrial Fibrillation; COR-AT, Safety and Efficacy of Vanoxerine for Conversion of Atrial Fibrillation or Flutter to Normal Sinus Rhythm; DIAGRAF-IKUR, Double-blind, International study AssessinG efficacy of S 066913 in paRoxysmal Atrial Fibrillation-IKur inhibitor; DOCTOS, DOxapram Conversion TO Sinus rhythm study; EAST-AFNET 4, Early Treatment of Atrial Fibrillation for Stroke Prevention Trial; SAFIRE-D, Symptomatic AF Investigative Research on Dofetilide; TREAT-AF, Transcutaneous Electrical Vagus Nerve Stimulation to Suppress Atrial Fibrillation.

## Abbreviations

**Abbreviations:** ACC, American College of Cardiology; ADT, antiarrhythmic drug therapy; AF, atrial fibrillation; AHA, American Heart Association; CaMKII, Ca^2+^/calmodulin kinase type II; CCB, Ca^2+^ channel blocker; CFAEs, complex fractionated atrial electrograms; dV/dt_max_, maximum upstroke velocity; ECGI, electrocardiographic imaging; ERP, effective refractory period; ESC, European Society of Cardiology; FDA, Food and Drug Administration; FIRM, focal impulse and rotor modulation; G_s_, stimulatory G-protein; hERG, human *ether-a-go-go*-related gene; HFpEF (HFrEF), heart failure with preserved (reduced) ejection fraction; I_CaL_, L-type Ca^2+^ current; I_f_, funny current; I_K1_, inward rectifier current; I_K2P_, two-pore-domain K^+^ current; I_KACh_, acetylcholine-activated inward-rectifier K^+^ current; I_Kr_/I_Kur_, rapid/ultrarapid delayed rectifier K^+^ current; (late) I_Na_, (late) Na^+^ current; I_SK_, small-conductance Ca^2+^-activated K^+^ current; I_ss_, steady-state current; LVEF, left ventricular ejection fraction (LVEF)LGE-MRI, late-gadolinium enhancement magnetic resonance imaging; LLTS, low-level tragus electrical stimulation; LLVS, low-level vagus nerve stimulation; miRNA, microRNA; Nav1.5, voltage-gated sodium channel; NCX, Na^+^-Ca^2+^ exchanger; NF-κB, nuclear factor κ-light-chain-enhancer of activated B cells; RAAS, renin-angiotensin-aldosterone system; RyR2, ryanodine receptor; SERCA2a, sarcoplasmic reticulum Ca^2+^-ATPase 2a; siRNA, small interfering RNA; SK, small-conductance Ca^2+^-activated K^+^ channel; TASK-1/-3, two-pore-domain Weak Inward rectifying K^+^ channel (TWIK)-related Acid-*S*ensitive K^+^ channel-1/-3.
